# Recent advances in understanding the role of sex hormone receptors in urothelial cancer

**DOI:** 10.32604/or.2025.062142

**Published:** 2025-05-29

**Authors:** MOHAMMAD AMIN ELAHI NAJAFI, TAKUO MATSUKAWA, HIROSHI MIYAMOTO

**Affiliations:** 1 Department of Pathology & Laboratory Medicine, University of Rochester Medical Center, Rochester, NY 14642, USA; 2 James P. Wilmot Cancer Institute, University of Rochester Medical Center, Rochester, NY 14642, USA; 3 Department of Urology, University of Rochester Medical Center, Rochester, NY 14642, USA

**Keywords:** Androgen receptor, Estrogen receptor, Bladder cancer, Urothelial cancer

## Abstract

Sex hormones, including androgens and estrogens, are known to have widespread physiological actions beyond the reproductive system via binding to their cognitive receptors, members of the nuclear receptor superfamily that function as ligand-inducible transcription factors. Meanwhile, a growing body of evidence has indicated the involvement of androgen receptor, as well as estrogen receptors such as estrogen receptor-α and estrogen receptor-β, in the pathogenesis and growth of various types of malignancies, including urothelial cancer. Additionally, in bladder cancer, the activity of sex hormone receptors has been implicated in modulating sensitivity to conventional non-surgical therapy. These may clearly explain sex-related differences in the incidence and prognosis of bladder cancer. This article focuses on summarizing the recent progress on understanding the role of sex hormones and their receptors in urothelial tumorigenesis, urothelial cancer progression, and resistance to non-surgical therapy for bladder cancer. Specifically, potential downstream effectors of sex hormone receptors have been newly identified. Thus, most of previous and subsequent data have indicated that activation of the androgen receptor or estrogen receptor-β pathway is favorable for urothelial cancer, while conflicting data exist especially on estrogen receptor-α functions.

## Abbreviation

ADT: Androgen deprivation therapy
AKR1C3: Aldo-keto reductase 1C3
AR: Androgen receptor
BBN: *N*-butyl-*N*-4-hydroxybutyl nitrosamine
BCG: Bacillus Calmette-Guérin
BPH: Benign prostatic hyperplasia
ChIP: Chromatin immunoprecipitation
CpdA: 2-(4-acetoxyphenyl)-2-chloro-*N*-methylethylammonium chloride
DHT: Dihydrotestosterone
E2: 17β-Estradiol
EGFR: Epidermal growth factor receptor
EMT: Epithelial-to-mesenchymal transition
ER: Estrogen receptor
ERK: Extracellular signal-regulated kinase
ERRα: Estrogen-related receptor alpha
FOXO1: Forkhead box protein O1
GATA3: GATA binding protein 3
GR: Glucocorticoid receptor
LPHN3: Latrophilin-3
MCA: 3-methylcholanthrene
MCM5: Minichromosome maintenance complex component 5
MMP: Matrix metalloproteinase
NF-κB: Nuclear factor-κB
mTOR: Mammalian target of rapamycin
PARP: Poly(ADP-ribose) polymerase
PBX1: Pre-B-cell leukemia transcription factor 1
PI3K: Phosphoinositide 3 kinase
SLPI: Secretory leukocyte peptidase inhibitor
STAT3: Signal transducer and activator of transcription 3
TCGA: The Cancer Genome Atlas
TUR: Transurethral resection
UGT1A: UDP-glucuronosyltransferase 1A

## Introduction

Urinary bladder cancer, which histologically shows urothelial carcinoma in most cases, has represented one of the most common malignancies, particularly among men, while the global numbers of newly diagnosed cases (e.g., 429,800 estimated in 2012 [1], 613,791 reported in 2022 [2]) and cancer-related deaths (e.g., 165,100 in 2012 [1], 220,349 in 2022 [2]) appear to be considerably increasing. Patients with bladder cancer often present with non-invasive disease but have a considerable risk of developing postoperative recurrence occasionally with invasive disease even after currently available intravesical pharmacotherapy. Moreover, muscle-invasive bladder cancer is often associated with metastatic disease where overall oncologic outcomes remain poor (e.g., 5-year survival rate of 8.8% [3]), even though new drugs for targeted therapy have been clinically employed [4,5]. In addition to the bladder (and urethra), urothelial carcinoma occurs in the upper urinary tract composed of the pyelocaliceal system and the ureter, which is much more often (e.g., approximately 60% [6]) invasive at the time of initial diagnosis. Accordingly, further identification of key molecules or signaling pathways and clarification of the molecular mechanisms responsible for urothelial tumorigenesis and urothelial tumor progression, which are believed to be distinct steps or events, may provide effective preventive and therapeutic strategies, respectively. Meanwhile, it is noteworthy to mention that there have been sex-related differences in the incidence (e.g., 3–4 times higher in men) and prognosis (e.g., mortality rate higher in women) of urothelial cancer [1,2,7].

Sex hormone receptors, including androgen receptor (AR), estrogen receptors (ERs) (e.g., ERα, ERβ), and progesterone receptors, are a group of steroid hormone receptors that are activated upon intracellular binding of their cognitive ligands, androgens, estrogens, and progestogens, respectively. These sex hormones and their receptors are known to mediate a variety of not only physiological processes but also pathologic conditions. Specifically, AR and ERα represent therapeutic targets in some endocrine-related malignancies, and anti-AR and anti-ER agents have been widely used for the treatment of, for example, prostate and breast cancers, respectively. Meanwhile, an increasing amount of evidence indicates the involvement of AR and ERs, as intrinsic factors, in the development and outgrowth of urothelial cancer, as well as the modulation of sensitivity to conventional non-surgical treatments for bladder cancer, such as intravesical bacillus Calmette-Guérin (BCG) immunotherapy, chemotherapy, and radiotherapy, although conflicting findings exist (reviewed in [8–10]). These may be helpful for clearly explaining some of the sex disparities in urothelial cancer.

We herein performed a computerized bibliographic search of the PubMed database, using the following keywords variably combined: “androgen”; “antiandrogen”; “antiestrogen”; “bladder cancer”; “estrogen”; “receptor”; “therapeutic resistance”; “therapy”; and “urothelial cancer”. We then selected only original studies published in peer-reviewed journals (plus some articles found in their reference lists). In this article, we thus summarize recent observations indicating the functional role of sex hormone receptors in urothelial cancer by focusing on the updates that have been documented in the last several years but may not have been covered in previous review articles with similar topics.

### Expression of AR in urothelial tumor and its prognostic significance

Studies assessing the status of AR expression in surgical specimens, mostly using immunohistochemistry, demonstrated that non-neoplastic urothelial cells were typically (e.g., >80% of cases) positive for AR and that AR expression was often down-regulated in bladder or upper urinary tract tumors (e.g., 11%–55%) [8,11,12]. Nonetheless, at least two studies [13,14] failed to detect AR immunoreactivity in any of “non-tumor” or “normal” tissues, although it was unclear if the expression in urothelial cells, but not stromal cells, had been adequately assessed. Additionally, in a substantial number of these studies [8,10,12], the rates of AR positivity were shown to be significantly lower in high-grade (*vs*. low-grade) and muscle-invasive (*vs*. non-muscle-invasive) tumors, while the significant down-regulation was confirmed only in high-grade tumors, but not in muscle-invasive tumors, in a meta-analysis in 2017 [11]. The prognostic impact of AR expression has also been assessed, but there are conflicting data indicating positive, negative, or no associations between AR expression and oncologic outcomes [8,10,12]. Specifically, the meta-analysis of 3 studies demonstrated an association of AR positivity in non-muscle-invasive tumors with a significantly lower risk of postoperative recurrence (hazard ratio 0.593, *p* = 0.006) [11]. These inconsistent data on the rate of AR expression and its associations with tumor grades/stages or prognosis among studies might have resulted from differences or lack of standardization in, for example, tissue preservation (e.g., timing of fixation), staining protocol (e.g., antibody), and immunoreactivity scoring.

An immunohistochemical study in 23 patients (16 males, 7 females) undergoing random biopsy showing no dysplastic changes compared the levels of AR expression in various regions in the non-neoplastic bladder (and prostatic urethra in men) [15]. AR levels were not significantly different among the regions including the trigone, posterior wall, anterior wall, dome, right wall, left wall, and prostatic urethra. In addition, there were no significant differences between men and women at each location, except that in the posterior wall [H-score: 94.4 (male) *vs*. 56.7 (female); *p* = 0.036]. Meanwhile, to the best of our knowledge, no studies have demonstrated the differences in AR expression in bladder tumors resected from various regions. Moreover, significant differences in AR expression between urothelial tumors from male *vs*. female patients have never been documented in previous studies, including more recent ones described below [16–21].

*AR* mRNA expression has been assessed in bladder cancer tissues. The levels of *AR* expression were significantly higher in non-muscle-invasive tumors (*n* = 29) than in muscle-invasive tumors (*n* = 12), while their difference between grade 2 (*n* = 10) and grade 3 (*n* = 31) tumors was not significant [16]. In another study involving 95 patients with high-risk (i.e., grade 3 and/or pT1) non-muscle-invasive bladder tumor, *AR* overexpression was associated with significantly better recurrence-free survival or cancer-specific survival [17]. *AR* expression in 252 cases with muscle-invasive bladder cancer additionally assessed via The Cancer Genome Atlas (TCGA) database in the former study [16] was strongly associated with TCGA molecular subtypes (e.g., lower in the basal subtypes than in the luminal subtypes). Furthermore, *AR* expression, as an independent prognosticator, was found to be associated with significantly worse disease-free survival in female patients, but not in male patients.

Five additional studies have immunohistochemically determined the expression of AR in 40 [18], 88 [19], 71 [20], 60 [21], and 132 [22] cases of bladder cancer. In contrast to the previous findings [8,10–12], the significant up-regulation of AR expression in high-grade or higher pT stage tumors was observed in one of them [19], whereas other three failed to show strong associations of AR expression with tumor grade or stage [18,20,22]. In addition, one described no AR expression at all in 20 cases of “non-neoplastic lesions” [20]. Moreover, AR expression was associated with significantly better [18] or worse [19] oncologic outcomes. Meanwhile, nuclear expression of AR has primarily been evaluated in all of previous and more recent immunohistochemical studies. However, a recent study showed that strong expression of cytoplasmic AR was significantly more often detected in non-muscle-invasive tumors (76.2%) than in muscle-invasive tumors (30.8%) [21]. Thus, the clinical impact of AR expression in urothelial tumors remains inconclusive.

### Expression of ERs in urothelial tumor and its prognostic significance

There are at least two intracellular ERs as nuclear receptors, ERα and ERβ. The overall rates of ERα and ERβ expression in urothelial tumors in previous immunohistochemical studies ranged from 0%–38% and 27%–100%, respectively [9]. Similar to AR data, conflicting results exist as to the differences in ERα and ERβ expression in non-neoplastic urothelial tissues *vs*. urothelial tumors, as well as low-grade or non-muscle-invasive *vs*. high-grade or muscle-invasive tumors. Specifically, in these comparisons, up-regulation, down-regulation, and no considerable change in ERα or ERβ expression in tumors (*vs*. non-tumors) and high-grade muscle-invasive tumors have been documented [9,12]. In a meta-analysis in 2017 [11], ERα expression was found to be significantly down-regulated in tumors, while ERβ expression was significantly up-regulated in high-grade or muscle-invasive tumors. There have been no studies demonstrating the prognostic value of ERα expression. ERβ positivity has been implicated in significantly worse and better patient outcomes, while other studies failed to show such strong associations [9,12]. Nonetheless, the meta-analysis revealed significant and marginal associations of ERβ positivity in non-muscle-invasive tumors with the risks of postoperative recurrence (hazard ratio 1.573, *p* = 0.013) and progression (hazard ratio 4.148, *p* = 0.089), respectively. Some issues in immunohistochemical studies described above (e.g., timing of fixation, different staining protocols and scoring systems) might explain the discrepancies in ERα/ERβ staining results. In particular, it is well known that delayed fixation after specimen collection (e.g., >60 min) leads to false-negative results in ERα immunohistochemistry in, for example, breast tissues [23]. More problematically, it has been indicated that the specificity of ER antibodies is not high and that only two of 13 commercially available anti-ERβ antibodies examined specifically target ERβ in immunohistochemical staining [24]. Moreover, one used to show ERβ immunoreactivity in all 313 bladder tumors was found even not to target ERβ [24,25].

The expression of *ESR1* and *ESR2* genes (encoding ERα and ERβ, respectively) was thereafter determined in bladder cancer specimens and its prognostic significance was assessed [26,27]. In 80 pT1 cases, *ESR2* overexpression was associated with a significantly higher risk of recurrence (*p* = 0.003) or progression (*p* = 0.044) in a univariate setting, but not in a multivariate setting, while *ESR1* overexpression tended to be associated with the risk of progression (*p* = 0.060) [27]. In 54 patients with muscle-invasive disease subsequently undergoing radical cystectomy, overexpression of all three genes examined, including *ESR1*, *ERBB2*, and KRT20 (54.3% *vs*. 15.8%; *p* = 0.009), but not *ESR1* alone (55.6% *vs*. 33.3%; *p* = 0.148), was significantly more often associated with complete response to neoadjuvant chemotherapy [26]. There was no significant difference in the levels of *ESR1* expression in the tumors obtained before [i.e., transurethral resection (TUR)] *vs*. after (i.e., cystectomy) neoadjuvant therapy [26].

Three additional immunohistochemical studies were performed in 132 [22], 115 [28], and 113 [29] cases of bladder tumors from TUR or cystectomy. In these studies, ERα or ERβ positivity in cancer cells was not strongly associated with tumor grades and/or stages. Instead, in 80 bladder tumors (unrelated to schistosomiasis), ERα expression in stromal cells was significantly (*p* = 0.032) more often detected in non-muscle-invasive cases (32%) than in muscle-invasive cases (12%) [28]. In addition, the rate of ERα positivity in tumor cells was slightly higher in bladder cancers associated with schistosomiasis known to produce estrogen-like metabolites (8/27, 30%) than in schistosomiasis-unrelated tumors (14/80, 18%) [28]. Moreover, in a study [22], patients with ERβ-positive non-muscle-invasive tumor were found to have a significantly (*p* = 0.046) lower risk of disease recurrence. Thus, the clinical impact of ERα and ERβ expression in urothelial tumors remains inconclusive. A study determining the immunoreactivity for estrogen synthesizing/metabolizing enzymes in bladder cancer specimens demonstrated that the positive rate of aromatase [pTa/pT1: 18/89 (20%) *vs*. pT2: 15/26 (58%)] or steroid sulfatase [63/89 (71%) *vs*. 9/26 (35%)] was significantly higher or lower, respectively, in muscle-invasive tumors than in non-muscle-invasive tumors [29].

### Impact of AR on urothelial tumorigenesis

The sex-specific differences in urothelial cancer, particularly male dominance in the incidence of bladder cancer [1,2], implied the involvement of AR signaling in urothelial tumorigenesis. A landmark study demonstrating no cancer development in the bladder of AR knockout mice treated with a strong chemical carcinogen *N*-butyl-*N*-4-hydroxybutyl nitrosamine (BBN) [30] indicated a critical role of AR in promoting urothelial carcinogenesis. Subsequent studies, using conditional AR knockout mice lacking AR only in urothelial cells [31] or transgenic mice conditionally expressing AR in the bladder urothelium [32], further indicated the impact of AR within urothelial cells, but not other cells such as those in the liver or kidney that could considerably affect the metabolism of carcinogens, on the development of bladder cancer. Correspondingly, androgen deprivation therapy (ADT) via chemical or surgical castration or anti-androgen treatment has prevented the development of bladder cancer in BBN-induced animal models [30,33]. Additionally, in an *in vitro* system where immortalized non-neoplastic urothelial SVHUC cells undergo neoplastic/malignant transformation induced by a chemical carcinogen 3-methylcholanthrene (MCA), AR overexpression or anti-androgen treatment resulted in the acceleration or retardation, respectively, of the neoplastic transformation [34,35]. Several retrospective cohort studies also supported the preclinical findings indicating the promotion of urothelial tumorigenesis by AR activation. These demonstrated significantly lower risks of: 1) subsequent development of bladder cancer in men with prostate cancer undergoing ADT than in those undergoing surgery alone or radiotherapy [36]; 2) postoperative recurrence of bladder cancer, especially AR-positive tumor, in men with both prostate and bladder cancers undergoing ADT for the treatment of the former than in those without ADT [37,38]; and 3) postoperative tumor recurrence in men with a history of bladder cancer undergoing ADT for their prostate cancer or 5α-reductase inhibitor dutasteride treatment for their benign prostatic hyperplasia (BPH) [39]. Moreover, a prospective study involving 72,370 men demonstrated that treatment with 5α-reductase inhibitor finasteride was associated with a significantly reduced risk of developing bladder cancer (hazard ratio 0.634, *p* = 0.0004) [40], although finasteride did not prevent the development of BBN-induced bladder tumor in an animal model [33]. Further studies demonstrated potential downstream targets of AR signaling that could modulate urothelial tumorigenesis. These included tumor suppressors down-regulated and/or inactivated by AR, including CD24 [41], GATA binding protein 3 (GATA3) [34], p53 [31], and UDP-glucuronosyltransferase 1A (UGT1A) [42], as well as oncogenic molecules up-regulated and/or activated by AR, including ATF2 [43], c-fos [44], ETS transcription factor ELK1 [44], and nuclear factor-κB (NF-κB) [45]. Meanwhile, dihydrotestosterone (DHT) was shown to be able to induce the development of BBN-mediated bladder cancer in whole-body AR knockout male mice [30], suggesting the involvement of the androgen-mediated non-AR pathway in urothelial tumorigenesis.

Two recent retrospective studies were conducted primarily to assess the role of androgens in the development or recurrence of bladder cancer [46,47]. In the first study, the levels of serum testosterone were compared in 147 men with bladder cancer *vs*. 154 age-matched controls with no bladder cancer [47]. Testosterone levels were slightly higher in bladder cancer patients (353 ng/dL) than in control men (332 ng/dL), and multivariate logistic regression analysis showed that testosterone was independently associated with the occurrence of bladder cancer (hazard ratio 1.002, *p* = 0.017). In the other study, the rates of postoperative recurrence were compared in men with non-muscle-invasive bladder cancer treated with dutasteride (for their BPH for at least 12 months; *n* = 165) *vs*. without 5α-reductase inhibitors (*n* = 147) [46]. Dutasteride treatment was significantly associated with a lower risk of recurrence, a lower number of recurrences, and a lower risk of developing muscle-invasive disease at recurrence, and multivariate analysis showed that dutasteride therapy was an independent predictor of recurrence (hazard ratio 0.67, *p* = 0.009). In contrast to the findings in the second study with dutasteride [46], as well as an earlier prospective study involving finasteride therapy [40], yet in line with *in vivo* data using the BBN-induced bladder carcinogenesis model [33], all 5α-reductase inhibitors examined, including dutasteride, finasteride, and epristeride, failed to considerably prevent the neoplastic transformation of MCA-SHVUC cells [48].

Compound A [CpdA; 2-(4-acetoxyphenyl)-2-chloro-N-methylethylammonium chloride] is a unique steroid hormone modulator known to function as not only a glucocorticoid receptor (GR) ligand but also an AR antagonist [49]. Indeed, CpdA has been shown to inhibit the transcriptional activity of AR in SVHUC-AR cells [50]. Then, CpdA was found to prevent the neoplastic transformation of MCA-SVHUC-AR cells more strongly than a glucocorticoid prednisone but failed to prevent that of AR-negative/GR-knockdown MCA-SVHUC-GR-shRNA cells, suggesting the suppression of urothelial tumorigenesis by CpdA via both the AR and GR pathways [50]. Additionally, in MCA-SVHUC-AR cells, CpdA significantly reduced the expression of oncogenes, such as *c-fos*, *c-jun*, and c*-myc*, and significantly induced that of tumor suppressors, such as *p21*, *p27*, *p53*, *PTEN*, and *UGT1A*. In the BBN-treated male mouse model, the rate of bladder cancer development in the CpdA group (25%) was significantly lower than that in the control (100%), antiandrogen flutamide (50%), or prednisone (50%) group [50].

The effects of silodosin, a selective α_1_-adrenergic receptor antagonist which is prescribed for the symptomatic treatment of BPH, on urothelial tumorigenesis have been assessed [51]. In SVHUC-AR cells, silodosin considerably reduced the expression of ELK1 which had been shown to promote urothelial tumorigenesis [44]. Correspondingly, silodosin treatment during its process prevented the neoplastic transformation of SVHUC-AR cells mediated by MCA and reduced or induced the expression of oncogenes (e.g., *c-fos*, *NF-κB*) or tumor suppressor genes (e.g., *p27*, *PTEN*), respectively, in resultant cells. However, other α_1_-blockers clinically used, such as tamsulosin and naftopidil, did not show such inhibitory effects on the expression of ELK1 or the neoplastic transformation of urothelial cells.

Two downstream targets of AR involving urothelial tumorigenesis, forkhead box protein O1 (FOXO1) [52] and latrophin-3 (LPHN3) [53], have been newly identified. A transcription factor FOXO1 functions as a tumor suppressor and is inactivated via phosphorylation, resulting in activation of several proteins, including the phosphoinositide 3 kinase (PI3K)/AKT pathway. AR overexpression in SVHUC cells or androgen treatment in SVHUC-AR cells reduced the expression of FOXO1, as well as its transcriptional activity, and induced the expression of an inactivated form phospho-FOXO1. Then, using the *in vitro* system, FOXO1 knockdown or inhibitor (i.e., AS1842856) treatment considerably induced the neoplastic transformation of SVHUC cells exposed to MCA, while the effects of knockdown/inhibitor were modest in MCA-SVHUC-AR cells where FOXO1 expression was considerably down-regulated. In addition, injection of AS1842856 resulted in the significant promotion of the development of BBN-induced bladder tumors in male mice. These observations indicate that AR inactivates FOXO1 in non-neoplastic urothelial cells, resulting in the induction of its tumorigenesis. Immunohistochemical staining in TUR specimens further demonstrated that the rates of FOXO1/phospho-FOXO1 positivity were significantly (both *p* < 0.001) lower and higher, respectively, in bladder tumors (13%/57%) than in normal-appearing urothelial tissues (40%/24%) and that FOXO1 positivity, as an independent predictor (hazard ratio 0.128, *p* = 0.043), was associated with a higher risk of postoperative recurrence of non-muscle-invasive tumors. LPHN3 (encoded by the *ADGRL3* gene) is a G-protein-coupled receptor to which a spider venom latrotoxin is known to bind. Overexpression of LPHN3 in SVHUC cells or treatment of an androgen in SVHUC-AR cells resulted in considerable increases in the levels of LPHN3 and *ADGRL3* expression. Moreover, chromatin immunoprecipitation (ChIP) assay revealed the binding of AR to the promoter region of *ADGRL3* in non-neoplastic urothelial cells, indicating the direct regulation of LPHN3 expression by AR. Meanwhile, treatment with α-latrotoxin or another LPHN3 ligand FLRT3 during the process of the neoplastic transformation or knockdown of LPHN3 induced or reduced, respectively, the oncogenic activity of resultant MCA-SVHUC/MCA-SVHUC-AR cells. Correspondingly, α-latrotoxin/FLRT3 injection accelerated the development of BBN-mediated bladder tumors only in female mice where endogenous LPHN3 expression was supposed to be lower than that in male mice. In immunohistochemical staining in TUR specimens, LPHN3 expression was significantly up-regulated in bladder tumors, compared with non-neoplastic urothelial tissues (*p* = 0.028), and the expression of LPHN3 and AR in tumors was significantly correlated (*p* = 0.021). LPHN3 positivity was also marginally (*p* = 0.051) associated with the recurrence of non-muscle-invasive tumors. These findings indicate that LPHN3 as a direct downstream effector promotes urothelial tumorigenesis.

### Impact of ERs on urothelial tumorigenesis

The effects of estrogen/anti-estrogen on the development of bladder cancer were assessed in animal models. A selective ER modulator tamoxifen strongly prevented BBN-induced carcinogenesis in female mouse bladders, presumably via ERβ because no ERα was detected in any of the bladders examined before exposure to BBN [54]. In another model where a carcinogen arsenic was exposed *in utero*, bladder cancer was detected only in female mice postnatally treated with a synthetic estrogen diethylstilbestrol (3/33, 9%), but not in those with vehicle (0/34) or tamoxifen (0/35) treatment [55]. These suggest that estrogen induces urothelial tumorigenesis.

The impact of ERα [56] and ERβ [57], which may have different functions, on bladder carcinogenesis has further been assessed, using knockout mouse models with BBN exposure. ERα knockout female mice (13/16, 81%) had a significantly (*p* = 0.030) higher incidence of bladder cancer than wild-type littermates (13/28, 46%), while the difference in that in male mice (knockout 11/13, 85% *vs*. wild-type 17/27, 63%) was not statistically significant. Similarly, bladder cancer was significantly (*p* = 0.021) more often seen in urothelium-specific ERα knockout female mice (16/21, 76%) than in wild-type females (12/30, 40%). By contrast, ERβ knockout in male (6/9, 67%) or female (3/13, 23%) mice resulted in an insignificantly (*p* = 0.206) or significantly (*p* = 0.017) lower incidence of bladder cancer, compared with wild-type males (8/8, 100%) or females (9/12, 75%), respectively. Additionally, in the *in vitro* transformation system, ERα overexpression [56] and ERβ knockdown [57], as well as tamoxifen treatment [52], in SVHUC cells resulted in significant reduction in their oncogenic activity mediated by MCA. These findings suggest that ERα and ERβ have inhibitory and stimulatory functions, respectively, in urothelial tumorigenesis. However, 17β-estradiol (E2) treatment in ERα-negative/ERβ-positive SVHUC cells rather induced the expression of UGT1A, a group of phase II drug metabolism enzymes known to play an important role in detoxifying bladder carcinogens such as aromatic amines and metabolites from tobacco, implying the preventive effect of estrogen/ERβ [58]. Meanwhile, ovariectomy in female mice resulted in significant reduction in the expression levels of *Ugt1a* subtypes in their bladders [58].

Only two recent studies have assessed the functional role of ERα [59], as well as ERβ and a related molecule FOXO1 [52], in urothelial tumorigenesis. In parallel with AR data, estrogen treatment induced the expression of an inactivated form phospho-FOXO1 and reduced the transcriptional activity of FOXO1 in SVHUC cells, whereas ERβ knockdown showed the opposite effects. Then, in the *in vitro* system, the effects of AS1842856, a FOXO1 inhibitor, on the neoplastic transformation were more significant in MCA-SVHUC-ERβ-shRNA cells, where the activity of FOXO1 was supposed to be considerably higher than in MCA-SVHUC cells. In addition, injection of FOXO1 inhibitor significantly accelerated the development of BBN-induced bladder tumors in female mice. These findings suggest that estrogen inactivates FOXO1, as a downstream effector of ERβ, and thereby promotes the development of urothelial tumor [52]. In a more recent study [59], the impact of ERα/*ESR1* on the initiation of bladder cancer was explored. FGFR3, a receptor tyrosine kinase, represents the oncogene most frequently altered in human bladder cancer, particularly non-muscle-invasive tumor. A gain-of-function mutation within the *FGFR3* gene in a transgenic mouse model facilitated the development of low-grade papillary bladder cancer, while *ESR1* expression was significantly down-regulated, suggesting the involvement of ERα in urothelial tumorigenesis as a tumor suppressor.

### Impact of AR on urothelial cancer progression

As shown in various other types of non-neoplastic and malignant cells, androgens induce the expression, nuclear translocation, and transcriptional activity of AR in bladder cancer cells [30,41,60–62]. Similarly, using *in vitro* models, androgens have been shown to promote the proliferation, migration, and invasion of AR-positive bladder cancer cells, while anti-androgen treatment or AR knockdown results in their retarded growth [30,41,60–63]. Additionally, in animal studies using heterotopic or orthotopic xenograft models or transgenic mouse models for bladder cancer, surgical or medical castration, as well as treatment with anti-AR agents, impaired the growth of tumors [8,30,63–65]. Previous studies have also identified a variety of molecules or pathways that not only involve tumor progression and metastasis, including epithelial-to-mesenchymal transition (EMT) and angiogenesis, but also are modulated via AR signals in urothelial cancer cells. These included ATF2 [43], β-catenin and its downstream c-myc [61], CD24 [41], epidermal growth factor receptor (EGFR) family and its downstream AKT/extracellular signal-regulated kinase (ERK) [60], ELK1 and its downstream c-fos [62], matrix metalloproteinases (MMPs) [30,64,66], NF-κB [45], Slug [63], and vascular endothelial growth factor [30,66].

Subsequent studies have identified potential downstream targets of AR involving the progression of urothelial cancer. Specifically, androgen/anti-androgen treatment in AR-positive bladder cancer cells or AR overexpression/knockdown could modulate the expression and/or activity of those contributing to promoting tumor progression, including LPHN3 [67], secretory leukocyte peptidase inhibitor (SLPI) involving vasculogenic mimicry [68], circular RNAs (e.g., circARC1 [69], circFNTA [70]), and microRNAs (e.g., miR-125b-2-3p [69], miR-370-3p [70], miR-525-5p [68], miR-4736 [69]), as well as a tumor suppressor FOXO1 [52] and a RNA editing enzyme *ADAR2* gene [70]. Meanwhile, AR activation was shown to reduce the expression of CD44 known to involve cell-cell interactions and cell adhesion/migration [71], suggesting the protective role of AR in bladder cancer. In some of these studies, ChIP assay confirmed AR binding to the promoter regions of, for example, *ADAR2* [70], *ADGRL3* [67], and *CD44* [71] genes in bladder cancer cells, indicating the direct regulation of their expression by AR.

The role of an anti-AR agent enzalutamide has been linked to autophagy in bladder cancer cells [72]. Enzalutamide treatment increased the accumulation of autophagosomes and autolysosomes in the cytoplasm and the expression of autophagy-related genes (e.g., *AMPK*, *ATG5*, *LC3B*, *ULK1*) and proteins (e.g., ATG5, LC3-II/I, phospho-AMPKα), implying that autophagy could represent an underlying mechanism for enzalutamide resistance. In addition, concurrent treatment of enzalutamide and an autophagy inhibitor chloroquine showed a synergistic effect on cell proliferation and apoptosis.

The effects of α_1_-blockers used for the treatment of BPH on the growth of bladder cancer cells have been assessed [51]. Silodosin treatment resulted in significant reduction in the viability and migration of AR-positive bladder cancer cells in a dose-dependent manner, while inhibiting ELK1 expression. However, no inhibitory effects of silodosin in AR-negative cells and tamsulosin or naftopidil in AR-positive/AR-negative cells on the cell growth were seen. Additionally, a retrospective analysis in bladder cancer patients undergoing silodosin (*n* = 49), tamsulosin (*n* = 64), or naftopidil (*n* = 28) therapy for their BPH showed that the risks of disease progression of non-muscle-invasive (*p* = 0.035) or muscle-invasive (*p* = 0.028) tumors were significantly lower in the silodosin group than in the tamsulosin or naftopidil group [51], supporting the preventive effect of silodosin on bladder cancer progression.

In recent immunohistochemical studies in surgical specimens or analyses of publicly available datasets, positive or negative correlations of AR *vs*. potential downstream targets, which support preclinical data, have been documented. These include correlations of AR with *CD44* [71] or phospho-FOXO1 [52]. In addition, a long non-coding RNA, FAM83H-AS1, was shown to be up-regulated in bladder cancers, while AR induced its expression in prostate cancer cells [73].

The impact of 5α-reductase inhibitors on bladder cancer progression has recently been assessed. A retrospective study showed that incidence of muscle-invasive disease was significantly (*p* = 0.041) lower in bladder cancer patients receiving dutasteride (2/165, 7.7%) than in those without 5α-reductase inhibitor therapy for their BPH (9/147, 16.7%) [46], implying the suppression of tumor invasion by dutasteride. In line with these data, dutasteride was shown to significantly inhibit the viability/proliferation and migration of AR-positive bladder cells, particularly with concurrent testosterone treatment [74]. Nonetheless, similar inhibitory effects of dutasteride on the cell growth, as well as the expression of oncogenic proteins including β-catenin, Bcl-2, MMP2, MMP9, NF-κB, and p21, were seen in AR-negative bladder cancer lines [74], suggesting the involvement of the non-AR pathway. By contrast, in another study, all three 5α-reductase inhibitors tested, including dutasteride, finasteride, and epristeride, failed to show significant inhibition in the viability and migration of two AR-positive bladder cancer cell lines [48].

As seen in BBN-treated AR knockout mice where DHT induced bladder cancer development [30], a recent study [75] demonstrated preclinical evidence suggesting the presence of the DHT-mediated pathway other than that via classical AR. Indeed, such non-classical ARs have recently been isolated. DHT was shown to induce the migration and invasion of bladder cancer cell lines lacking the classical AR where a membrane AR, named mAR-SLC39A9 was expressed, while augmenting the expression of phospho-ERK and MMP9 only in those expressing SLC39A9 [76], indicating that DHT could promote bladder cancer progression via mAR-SLC39A9. The analysis of TCGA database then revealed that the expression of SLC39A9 was considerably elevated in bladder cancers (*vs*. non-tumors), which was further associated with significantly worse disease-free survival (*p* = 0.003) or overall survival (*p* = 0.008) [76]. Another group identified AR-v19, a novel splice variant of AR, in bladder cancer [77]. Silencing of AR-v19 in bladder cancer lines resulted in a decrease in cell viability and increases in apoptosis and the expression of related molecules, such as cleaved poly(ADP-ribose) polymerase (PARP) [77]. RNA-sequencing further indicated AR-v19-mediated regulation of various signaling pathways involving cell proliferation and cellular metabolism, including the mammalian target of rapamycin (mTOR) [78].

### Impact of ERs on urothelial cancer progression

Earlier studies [9,79–81], using bladder cancer lines and mouse xenograft models for bladder cancer and demonstrating E2-induced proliferation of ERα-positive cells and its inhibition by selective ER modulators, as well as inhibition of the growth of ERα-negative/ERβ-positive tumor by tamoxifen, indicated the stimulatory effects of estrogen on the progression of urothelial cancer. Both a selective ERα ligand and a selective ERβ ligand also induced the proliferation of ERα-positive/ERβ-positive cells [80], suggesting the similar function of ERα and ERβ on bladder cancer outgrowth. In addition, knockdown of ERβ or treatment with an ERβ-selective antagonist resulted in the inhibition of cell growth [57]. However, it was also documented that knockdown or overexpression of ERα induced or reduced, respectively, the growth of bladder cancer cells and xenograft tumors [56], suggesting the suppressive role of ERα in urothelial cancer. Thus, data are mostly consistent, indicating the promoting function of ERβ, but those of ERα are conflicting as to its role in the progression of urothelial cancer. Potential downstream targets/pathways of ER signaling in bladder cancer cells were also identified and included ERK (activated by E2 [80]), AKT (via ERα [56]), and minichromosome maintenance complex component 5 (MCM5) involving the initiation of DNA replication (via ERβ [57]). Previous studies have additionally indicated the involvement of ER signaling in the microenvironment of bladder cancer, such as cancer-associated immune cells (e.g., reduction of cancer cell invasion via modulating EMT and CCL2/CCR2/MMP9 signals by ERβ knockdown in co-cultured mast cells [82]) and cancer-associated fibroblasts (e.g., modulation of cancer cell invasion by ERα overexpression/knockdown in co-cultured fibroblasts [83]). Co-culture of CD4+ T-cells [84] or cancer-associated fibroblasts [85] with bladder cancer cells also induced ERβ expression. In addition to nuclear ERs, G protein-coupled estrogen receptor 1 (also known as GPR30) isolated as a membrane ER [86] was found to inhibit the growth of bladder cancer cells [87].

As seen in AR signals, ERβ has been linked to a tumor suppressor FOXO1, as a downstream target, in bladder cancer cells [52]. The activity of ERβ and FOXO1 was inversely correlated, and ERβ-mediated FOXO1 inactivation resulted in the promotion of bladder cancer cell proliferation and migration. Moreover, ChIP assay in bladder cancer cells revealed the binding of ERβ to the promoter region of *FOXO1*. These findings suggest that FOXO1 functions as a direct downstream effector of ERβ in urothelial cancer cells and prevents tumor growth. Meanwhile, in bladder cancer specimens, the expression of FOXO1 *vs*. ERα or ERβ was positively or negatively, respectively, correlated.

Pre-B-cell leukemia transcription factor 1 (PBX1), a transcription factor known to involve cancer cell proliferation, apoptosis, and EMT, has been implicated in ER signaling in bladder cancer [88]. Both ERα and ERβ were shown to interact with PBX1 in bladder cancer cells, which was required for ER function. Overexpression of PBX1 enhanced the stimulatory effect of estrogen on the cell growth and diminished the suppressive effect of a selective ER antagonist AZD9496.

Recent studies have also indicated that ERs regulate the expression of microRNAs and circular RNAs, both of which are known to contribute to bladder cancer progression, via direct binding to their promoters in bladder cancer cells. Specifically, ERα induced the expression of miR-4324 and thereby prevented tumor growth via signal transducer and activator of transcription 3 (STAT3) signaling [89]. Similarly, ERα reduced the levels of circ_0023642 expression, resulting in the induction of miR-490-5p expression and subsequent inhibition of EGFR signaling [90]. In addition, ERβ induced miR-92a expression and subsequently reduced the expression of a tumor suppressor DAB2IP [91]. These findings support the inhibitory role of ERα and the stimulatory role of ERβ in the progression of urothelial cancer.

The impact of estrogen-related receptor alpha (ERRα), a nuclear receptor sharing structural and functional properties with ERs, on bladder cancer has also been assessed [92]. Immunohistochemistry in bladder cancer specimens (*n* = 61) showed that ERRα expression was significantly more often detected in high-grade tumors, higher stage (i.e., stages II–IV) cases, or those with distant metastasis and was associated with significantly worse overall survival, as an independent prognosticator (hazard ratio 4.15, *p* = 0.015). In bladder cancer lines, silencing of ERRα resulted in significant reduction of the cell viability, migration, and invasion, and significant induction of apoptosis, while inhibiting EMT. ERRα silencing also inhibited the growth of xenograft tumors in mice. These observations suggest that ERRα promotes the progression of urothelial cancer.

### Impact of AR on modulating sensitivity to conventional non-surgical therapy

Previous studies suggested that AR activation induced resistance to conventional non-surgical therapy for bladder cancer [10], including intravesical BCG immunotherapy [93], intravesical or systemic chemotherapy [94–97], and radiotherapy [98]. Chemotherapeutic drugs in the studies clearly showing the association of their efficacy with AR activity for the first time were cisplatin [95], doxorubicin [94], gemcitabine [96], and mitomycin [97]. In addition, some downstream molecules, such as cyclin D1 [96], ELK1 [62], and NF-κB [45,95], have been shown to involve AR-induced chemoresistance. A selective α_1_-blocker silodosin, which could down-regulate ELK1 expression, thus increased cisplatin sensitivity in ELK1-positive bladder cancer cells [99]. Moreover, AR expression was found to be considerably elevated in bladder cancer sublines resistant to cisplatin [95] or gemcitabine [96] therapy, and radiotherapy [98]. Similarly, in TUR specimens, overexpression of AR [95], as well as phospho-ELK1 [99] and phospho-NF-κB [95], has been associated with resistance to cisplatin-based neoadjuvant chemotherapy prior to radical cystectomy. Accordingly, concurrent anti-AR therapy, apart from its direct anti-tumor activity, was anticipated to enhance the efficacy of conventional therapy in patients with urothelial cancer.

Additive inhibition of the growth of xenograft tumors heterotopically injected with murine bladder cancer MBT-2 cells by enzalutamide, together with intratumoral injection of BCG, was observed in male mice, potentially via modulating the immune cell composition within the tumor [100]. In a more mechanistic study [101], the involvement of Rab27b, a small GTPase known to enhance bacterial exocytosis, in the modulation of AR-mediated efficacy of BCG, attenuated form of the bacteria *Mycobacterium bovis*. In bladder cancer cells, AR activity was positively and inversely correlated with the expression of Rab27b and the amount of intracellular BCG, respectively. In an orthotopic mouse xenograft model with murine bladder cancer MB49 cells, knockdown of Rab27b or its effector SYTL3 in MB49 and concurrent treatment with an exocytosis inhibitor GW4609 in MB49-bearing mice similarly enhanced the anti-tumor effects of intravesical BCG therapy, while increasing the amount of BCG evaluated via acid-fast bacillus staining. These findings suggest that AR up-regulates Rab27b expression, resulting in the enhancement of BCG elimination from urothelial cells and resistance to BCG therapy. Indeed, immunohistochemistry in TUR specimens showed that patients with AR-positive or Rab27b-positive non-muscle-invasive bladder tumor subsequently undergoing BCG therapy had a significantly higher risk of postoperative disease recurrence, compared to those with AR/Rab27b-negative tumor. Thus, concurrent ADT may considerably enhance the efficacy of intravesical BCG immunotherapy particularly in men with AR-positive bladder cancer, while AR immunohistochemistry may be useful for predicting response to BCG therapy.

An increase or a decrease in cisplatin cytotoxicity via inactivation (e.g., anti-androgen treatment, AR knockdown) [70,102] or overexpression [103] of AR in bladder cancer cells has been confirmed in more recent studies. Potential downstream effectors of AR that modulate cisplatin sensitivity in bladder cancer have been additionally identified. Those up-regulated by AR include circFNTA [70] and a GABA B receptor GABBR2 [104], whereas others down-regulated or inactivated by AR are FOXO1 [105], BXDC2 [106], a core protein involving ribosome biogenesis, and GULP1 [107], an adaptor protein known to facilitate phagocytosis. Of these, AR has been shown to bind the promoter regions of *GABBR2* [104] and *BXDC2* [106]. Meanwhile, the impact of aldo-keto reductase 1C3 (AKR1C3), an androgen-metabolizing enzyme involving the production of DHT, on chemoresistance has been explored in bladder cancer [108]. A chromene derivative 2j, which could function as a selective inhibitor for AKR1C3, was found to inhibit the expression of AR and ELK1 in bladder cancer cells and enhance the cytotoxic activity of gemcitabine + cisplatin. Furthermore, in 58 patients with muscle-invasive bladder cancer, strong AKR1C3 expression, as an independent predictor, was associated with significantly worse oncologic outcomes after radical cystectomy.

Recent data suggest that AR signaling may also modulate the efficacy of immune checkpoint inhibitors used for the treatment of advanced urothelial cancer. In line with the findings in a previous immunohistochemical study in urothelial carcinoma of the bladder (*n* = 85) and upper urinary tract (*n* = 25) [109], strong inverse correlations between AR and PD-L1 expression in urothelial carcinoma cells were observed in nephroureterectomy specimens from 99 patients [110] and in a set of commercially available tissue microarray consisting of 103 bladder cancer cases [111], as well as in human bladder urothelial carcinoma cell lines [111]. However, in another recent study using TUR (*n* = 143) and radical cystectomy (*n* = 198) specimens [112], there were no strong correlations between AR expression in tumor cells and PD-L1 expression in tumor cells or tumor-associated immune cells. In addition, knockdown and overexpression of AR in bladder cancer lines resulted in an increase and a decrease, respectively, in the expression of PD-L1 [111]. Because the levels of PD-L1 expression may likely be associated with sensitivity to PD-1/PD-L1 blockade with immune checkpoint inhibitors, concurrent AR inhibition (e.g., ADT), which enhances PD-L1 expression, is anticipated to considerably increase its efficacy in patients with urothelial cancer. In a study using a xenograft model with murine MBT-2 cells in immunocompetent mice [100], additive (not synergistic) effects of PD-L1 antibody + enzalutamide were seen, but it was not clear if enzalutamide independently inhibited the tumor growth. Surprisingly, in another mouse xenograft study [111], anti-PD-L1 antibody more significantly inhibited the growth of MBT-2 xenografts stably overexpressing AR (where PD-L1 expression was considerably down-regulated based on the observations in the same study). Similarly, AR overexpression or knockdown/inactivation was also documented to induce or reduce, respectively, the expression of PD-L1 in bladder cancer cells where circ_0001005 and miR-200a-3p, which further enhanced the cytotoxicity of NK cells, were involved [113]. It thus remains controversial even if AR negatively *vs*. positively regulates PD-L1 expression in urothelial cancer cells.

### Impact of ERs on modulating sensitivity to conventional non-surgical therapy

Earlier studies in bladder cancer lines showed that tamoxifen treatment, along with methotrexate [114], vinblastine [114], doxorubicin [114,115], cisplatin [114], mitomycin C [115], or gemcitabine [116], more strongly inhibited the cell growth, but the combination effects were not adequately compared. Therefore, it could not conclude that tamoxifen indeed enhanced sensitivity to each chemotherapeutic agent in bladder cancer cells. No studies appear to have assessed the role or ERs in modulating sensitivity to immunotherapy in urothelial cancer.

A subsequent study demonstrated that co-culture of cancer-associated fibroblasts, while inducing ERβ expression in bladder cancer cells as described above, also reduced the cytotoxic activity of cisplatin [85]. In a more recent study [117], not only tamoxifen treatment but also ERβ knockdown in ERα-negative bladder cancer lines resulted in significant increases in sensitivity to cisplatin therapy. By contrast, E2 treatment in ERα-negative/ERβ-positive bladder cancer cells significantly reduced the cisplatin cytotoxicity. Moreover, ERβ expression was found to be considerably elevated in cisplatin-resistant bladder cancer sublines (*vs*. respective control sublines), and immunoreactivity for ERβ was significantly more often detected in bladder tumors from patients who had not responded well to subsequent neoadjuvant cisplatin-based chemotherapy (22/31, 71%) than in those from responders (9/24, 38%) [117]. These findings clearly indicate that ERβ signaling induces cisplatin resistance in bladder cancer. Meanwhile, only limited data are available for the role of ERα in modulating chemosensitivity. In a study [89], ERα was shown to induce the expression of miR-4324 via binding to its promoter, while miR-4324 enhanced the cytotoxic activity of doxorubicin in bladder cancer cells, suggesting the favorable effects of ERα. As aforementioned, however, the expression of *ESR1* alone in TUR specimens from those with muscle-invasive bladder cancer was not strongly associated with complete response to neoadjuvant chemotherapy [26].

Potential downstream effectors of ERβ have also been identified in bladder cancer. ERβ was found to inactivate FOXO1 [105] and reduce the expression of GULP1 [118] via binding to their promoters in bladder cancer cells and thereby induce sensitivity to cisplatin. In addition, E2 treatment in ERα-negative/ERβ-positive bladder cancer cells induced the expression and activity of β-catenin [117] known to contribute to cisplatin resistance. Overexpression of PBX1, which could interact with both ERα and ERβ, was also shown to reduce the cisplatin cytotoxicity in bladder cancer cells [88].

## Conclusive Remarks and Therapeutic Prospects

**Figure 1:**
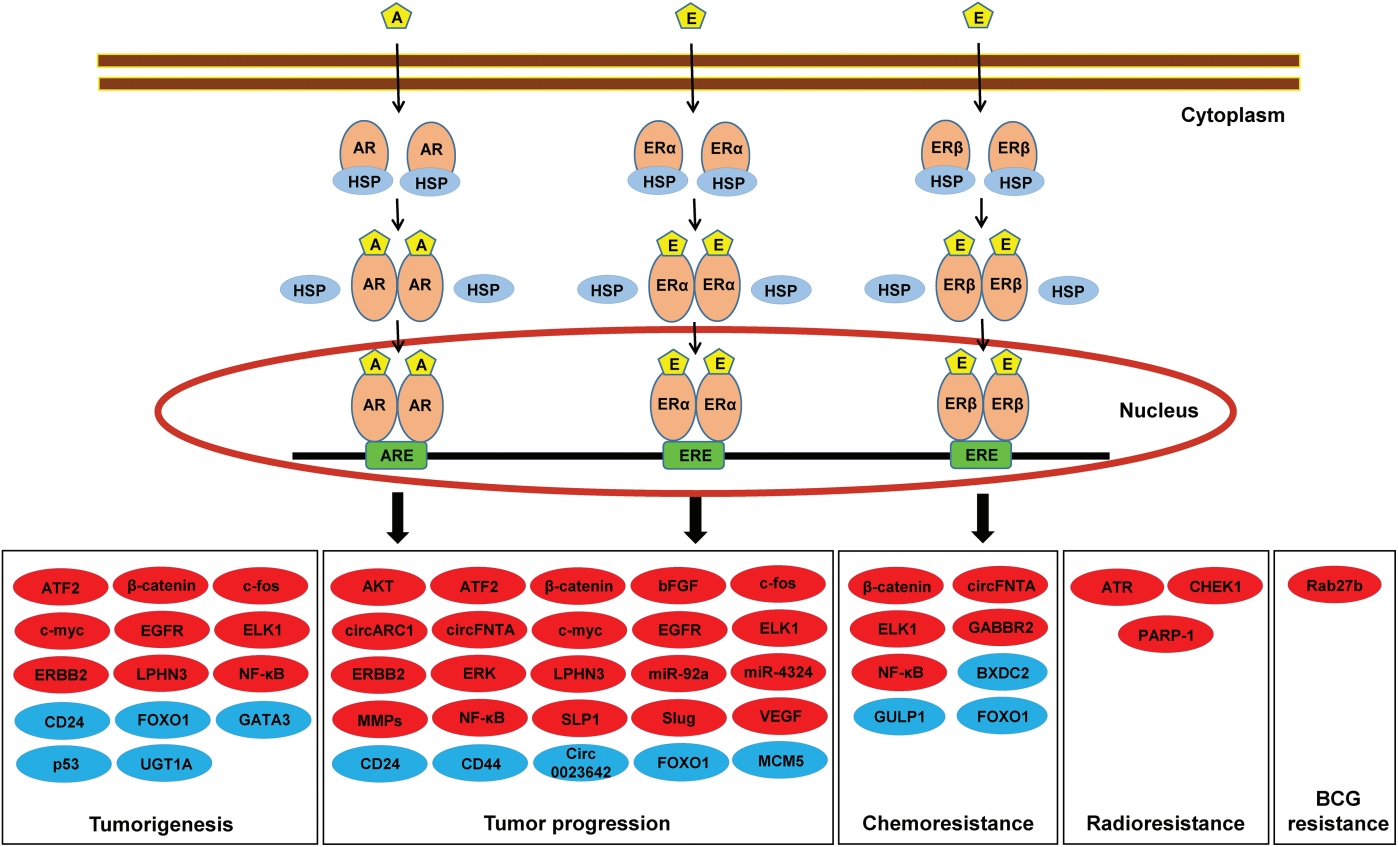
Androgen/estrogen-mediated signals, including potential downstream effectors, in non-neoplastic urothelial cells or urothelial cancer cells. Androgens or estrogens have been suggested to modulate tumorigenesis and tumor progression, as well as resistance to conventional non-surgical therapy for bladder cancer, through the ligand-mediated AR, ERα, and/or ERβ pathway(s) via up-regulating/activating (red) or down-regulating/inactivating (blue) the downstream targets listed. A, androgen; ARE, androgen response element; E, estrogen; ERE, estrogen response element; HSP, heat shock protein.

Available evidence indicates a critical role of sex hormone receptor signaling in the development and progression of urothelial cancer, as well as the modulation of sensitivity to conventional non-surgical therapy for bladder cancer. Specifically, most of previous and subsequent studies have demonstrated that activation of AR and ERβ is favorable for urothelial cancer for its tumorigenesis and outgrowth, and is implicated in therapeutic resistance. However, conflicting data, especially those on the functions of ERα, exist. Fig. 1 and Table 1 summarize the molecules shown to be up-regulated/down-regulated and/or activated/inactivated by AR, ERα, and/or ERβ in non-neoplastic urothelial cells and/or urothelial cancer cells, as potential effectors. Meanwhile, besides some immunohistochemical studies in upper urinary tract urothelial carcinoma specimens showing the results similar to those in bladder cancer specimens [109,110,119], no studies appear to have assessed the role of AR, ERα, and/or ERβ specifically in upper urinary tract cancer. Thus, it remains uncertain if their roles in the development and progression of urothelial cancer differ between bladder and upper urinary tract cancers.

**Table 1:** Potential downstream targets of androgen and/or estrogen signals

Molecule [Reference]	Related receptor	Resultant changes in non-neoplastic urothelial or urothelial cancer cells	Subsequent impact on urothelial cancer
AKT [56,60]	AR ERα	Activation	Growth↑
ATF2 [43]	AR	Upregulation Nuclear translocation Activation	Tumorigenesis↑ Growth↑
ATR [98]	AR	Upregulation	Radioresistance↑
β-catenin [61,117]	AR ERβ	Nuclear translocation Activation	Tumorigenesis↑ Growth↑ Chemoresistance↑
Basic fibroblast growth factor (bFGF) [30]	AR	Upregulation	Growth↑
BXDC2 [106]	AR	Downregulation	Chemoresistance↑
CD24 [41]	AR	Downregulation	Tumorigenesis↑ Growth↑
CD44 [71]	AR	Downregulation	Growth↓
c-fos [44,62]	AR	Upregulation	Tumorigenesis↑ Growth↑
Checkpoint kinase 1 (CHEK1) [98]	AR	Upregulation	Radioresistance↑
Circ 0023642 [90]	ERα	Downregulation	Growth↓
circARC1 [69]	AR	Upregulation	Growth↑
circFNTA [70]	AR	Upregulation	Growth↑ Chemoresistance↑
c-myc [61]	AR	Upregulation	Tumorigenesis↑ Growth↑
EGFR [60]	AR	Upregulation Activation	Tumorigenesis↑ Growth↑
ELK1 [44,62,99]	AR	Upregulation Nuclear translocation	Tumorigenesis↑ Growth↑ Chemoresistance↑
ERBB2 [60]	AR	Upregulation Activation	Tumorigenesis↑ Growth↑
ERK [60,76,80]	AR mAR-SLC39A9 ER	Activation	Growth↑
FOXO1 [52,105]	AR ERβ	Downregulation Inactivation	Tumorigenesis↑ Growth↑ Chemoresistance↑
GABBR2 [104]	AR	Upregulation	Chemoresistance↑
GATA3 [34]	AR	Downregulation	Tumorigenesis↑
GULP1 [107,118]	AR ERβ	Downregulation	Chemoresistance↑
LPHN3 [53,67]	AR	Upregulation	Tumorigenesis↑ Growth↑
MCM5 [57]	ERβ	Downregulation	Growth↑
miR-92a [91]	ERβ	Upregulation	Growth↑
miR-4324 [89]	ERβ	Upregulation	Growth↓
MMPs [30,64,66,76]	AR mAR-SLC39A9	Upregulation	Growth↑
mTOR [78]	AR-v19	Upregulation	Growth↑
NF-κB [45,95]	AR	Upregulation Nuclear translocation Activation	Tumorigenesis↑ Growth↑ Chemoresistance↑
p53 [31]	AR	Downregulation	Tumorigenesis↑
PARP-1 [97]	AR	Upregulation	Radioresistance↑
Rab27b [101]	AR	Upregulation	BCG exocytosis↑
SLPI [68]	AR	Upregulation	Growth↑
Slug [63]	AR	Upregulation	Growth↑
UGT1A [42,58]	AR ERβ	Downregulation	Tumorigenesis↑
VEGF [30,66]	AR	Upregulation	Growth↑

Based on the observations using preclinical models and surgical specimens as well as those from retrospective clinical studies, early phase clinical trials have been conducted to assess the efficacy of available hormonal therapy agents in patients with urothelial cancer (Table 2). In a phase 1/1b trial (NCT02300610) primarily assessing the safety and tolerability of enzalutamide in combination with gemcitabine and cisplatin in a total of 10 patients with metastatic bladder cancer, complete response was achieved in a patient with strongly AR-positive tumor [120]. In a phase 2 trial assessing the effects of enzalutamide on preventing the post-TUR recurrence of non-muscle-invasive bladder tumor (NCT02605863), the recruitment of the patients did not appear to be successful. Other completed phase 2 studies have assessed the efficacy of tamoxifen (NCT00710970, NCT02197897) or genistein (NCT00118040), a phytoestrogen with structure similar to that of E2, in patients with bladder cancer, but no results appear to have been documented in the literature. In addition, participants are being recruited or prepared for the recruitment in several phase 1 or 2 studies that assess the efficacy of: 1) an AR antagonist bicalutamide in those with non-muscle-invasive bladder tumor eligible for intravesical BCG therapy (NCT05327647); 2) an AR antagonist apalutamide via determining the expression of an AR target EGFR in non-muscle-invasive bladder tumors (NCT05521698); and 3) degarelix, a gonadotropin-releasing hormone antagonist used as chemical castration, in those with muscle-invasive bladder cancer undergoing systemic chemotherapy with gemcitabine and cisplatin (NCT05839119). Overall, current clinical trial data are insufficient or too preliminary and neither support nor contrast preclinical evidence indicating important roles of AR or ER signaling in urothelial cancer.

**Table 2:** Clinical trials involving androgen or estrogen signals in patients with bladder cancer in the US

ID	Phase	Main inclusion criteria (*N* recruited)	Intervention/treatment	Main outcomes	Status	Ref.
NCT00118040	II	One tumor, candidates for TUR or cystectomy (*N* = 60)	Genistein (daily for 14–30 days)	Reduced pEGFR strength signal	Completed (Aug 2010)	NA
NCT00710970	II	Metastatic disease with 1–2 systemic therapy regimen (*N* = 28)	Tamoxifen (daily)	NA	Completed (Dec 2012)	NA
NCT02605863	II	NMIBC after TUR (*N* = 1)	Enzalutamide (daily for 12 months)	NA	Terminated (Feb 2018)	NA
NCT02300610	I/Ib	Stage IV (*N* = 10)	Enzalutamide (daily) + GC (6 cycles)	Complete response in 1 patient	Completed (Apr 2019)	120
NCT02197897	II	Low/intermediate risk papillary 6–10 mm UC (*N* = 15)	Tamoxifen (daily for 12 weeks)	NA	Completed (Jun 2019)	NA
NCT05327647	II	NMIBC eligible for intravesical BCG therapy	BCG + Bicalutamide (daily for 90 days)	NA	Recruiting	NA
NCT05521698	I	NMIBC	Apalutamide (up to 28 days) → TUR	NA	Not yet recruiting	NA
NCT05839119	I	MIBC prior to cystectomy	Degarelix + GC (4 cycles)	NA	Recruiting	NA

Note: EGFR, epidermal growth factor receptor; GC, gemcitabine + cisplatin; MIBC, muscle-invasive bladder cancer; NA, not available; NMIBC, non-muscle-invasive bladder cancer; TUR, transurethral resection; UC urothelial carcinoma.

Remarkably, androgen or estrogen ablation has been widely used for the treatment of, for example, prostate or breast cancer, respectively, and various drugs and therapeutic options are clinically available. It may therefore be able to be readily applied to any of these to urothelial cancer patients. Further clinical studies, including prospective cohort trials, for hormonal therapy are thus encouraged. Specifically, hormonal modification is expected to contribute to effective prevention of the postoperative recurrence of non-muscle-invasive bladder cancer and suppression of the growth of invasive urothelial cancer and its metastasis, particularly AR-positive or ERβ-positive tumors. In addition to its direct anti-tumor effects, concurrent anti-AR or anti-ERβ therapy is also expected to be beneficial for enhancing sensitivity to conventional non-surgical treatments for bladder cancer, particularly cisplatin therapy in men with AR-positive tumor or in women with ERβ-positive tumor, respectively. Meanwhile, loss of AR (or ERβ) in tumor specimens may serve as an indicator of high sensitivity to these non-surgical treatments. Suppression of the downstream targets of AR/ERβ up-regulated/activated and promotion of those down-regulated/inactivated may also provide novel targeted therapy via specific inhibitors and activators, respectively, if they are available but have not been applied to urothelial cancer therapy.

In conclusion, it remains far from being fully understood how sex hormones and related receptors function in non-neoplastic urothelial cells where the neoplastic transformation is initiated, as well as in urothelial cancer cells. Additional investigation is still warranted to precisely determine the biological functions of androgens and estrogens in urothelial cells and elucidate underlying molecular mechanisms for their actions via the AR/ER pathways.

## Data Availability

Data sharing is not applicable to this article as no datasets were generated or analyzed during the current study.
